# Supplementary figures and images for: Acute Effect of a Saffron Extract (Safr’InsideTM) and Its Main Volatile Compound on the Stress Response in Healthy Young Men: A Randomized, Double Blind, Placebo-Controlled, Crossover Study
Source: Nutrients. 2023 Jun 27;15(13):2921. doi: 10.3390/nu15132921 (PMC10346743; doi:10.3390/nu15132921)

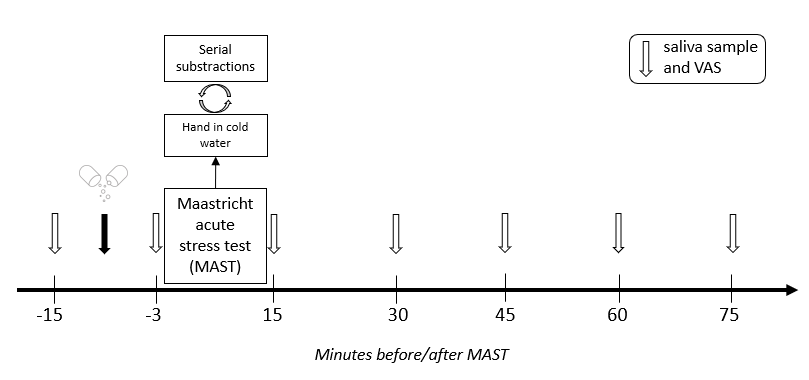

Supplement: Supplementary file 1 [file nutrients-15-02921-s001.zip › nutrients-2443525-supplementary.PNG]
